# Risk of vertebral compression fractures in multiple myeloma patients: A finite-element study

**DOI:** 10.1097/MD.0000000000005825

**Published:** 2017-01-13

**Authors:** D. Anitha, Baum Thomas, Kirschke S. Jan, Karupppasamy Subburaj

**Affiliations:** a Engineering Product Development (EPD), Singapore University of Technology and Design (SUTD), Singapore; b Department of Radiology, Klinikum rechts der Isar, Technische Universitaet Muenchen, Muenchen, Germany.

**Keywords:** finite element analysis, MDCT, multiple myeloma, vertebral fracture

## Abstract

The purpose of this study was to develop and validate a finite element (FE) model to predict vertebral bone strength in vitro using multidetector computed tomography (MDCT) images in multiple myeloma (MM) patients, to serve as a complementing tool to assess fracture risk. In addition, it also aims to differentiate MM patients with and without vertebral compression fractures (VCFs) by performing FE analysis on vertebra segments (T1–L5) obtained from in vivo routine MDCT imaging scans. MDCT-based FE models were developed from the in vitro vertebrae samples and were then applied to the in vivo vertebrae segments of MM patients (n = 4) after validation. Predicted fracture load using FE models correlated significantly with experimentally measured failure load (*r* = 0.85, *P* < 0.001). Interestingly, an erratic behavior was observed in patients with fractures (n = 2) and a more gradual change in FE-predicted strength values in patients without fractures (n = 2). Severe geometric deformations were also observed in models that have already attained fractures. Since BMD is not a reliable parameter for fracture risk prediction in MM subjects, it is necessary to use advanced tools such as FE analysis to predict individual fracture risk. If peaks are observed between adjacent segments in an MM patient, it can be safe to conclude that the spine is experiencing regions of structural instability. Such an FE visualization may have therapeutic consequences to prevent MM associated vertebral fractures.

## 1 Introduction

Multiple myeloma (MM) primarily occurs in the elderly, with the median diagnosis reported at 69 years. As per the National Cancer Institute report, MM comprises of 1.6% of all bone malignancies in the United States and the 5-year survival rate is less than 50%.^[1]^ Although often misunderstood as a rare disease, MM is in fact the 2nd most commonly diagnosed hematologic malignancy in the Western world.^[2]^ Being a skeletal malignant disease of older adults, this disease is a clinically significant problem, if left unresolved. This is mainly attributed to a substantial increase in life expectancy with medical advancements, resulting in a continually increasing aged population, shifting the bulk of socioeconomic burden onto elderly care services and facilities.^[3]^ Vertebral compression fractures (VCFs) are the most common type of fractures in patients with MM.^[4]^ VCFs are known to occur at the onset of MM in 34% to 64% of patients.^[5]^ The efficacy of current intervention therapies on reducing the risk of mortality remains less well understood.^[6]^ Accordingly, early diagnosis and treatment are critical in slowing down the disease progression rate and deterioration of quality of life.^[7]^

Most MM patients report back pain at diagnosis.^[8]^ Although MM patients are checked for biological symptoms such as anemia, hypercalcemia, or renal insufficiency,^[9]^ radiological indications for the presence of MM are diffuse bone loss, focal osteolytic bone lesions and bone marrow edema, and fragility axial fractures. Diffuse bone loss alone is often misdiagnosed as Osteoporosis, a skeletal disease known to result in significant bone loss,^[10]^ until more symptoms associated with MM develops. Bone marrow edema is a common finding during magnetic resonance imaging in acute VCFs.^[11]^ Majority of MM cases, as high as 80% of patients, are diagnosed during routine radiological scan procedures.^[12]^ When focal osteolytic lesions or significant diffuse bone loss are observed, risk for axial fractures in the vertebrae are high.^[13]^

Bone mineral density (BMD) and its related T-score are the indicators for assessing the risk of fracture. Almost 80% of MM patients are diagnosed with osteoporosis; hence BMD currently has a major impact on survival in MM patients.^[14,15]^ It is believed that osteoporosis status could be an indicator of disease progression to MM.^[16]^ Osteoporotic patients (20%) presenting with vertebral fractures have either monoclonal gammopathy undetermined significance or MM.^[13]^ However, there are 3 problems with the use of BMD as a diagnostic tool of MM. First, decline in BMD as a result of aging and/or osteoporosis has been understood; it declines 0.1% to 0.2% per year due to aging while after menopause and onset of osteoporosis, it peaks to 1% to 2% and then slows back to decline due to aging.^[9]^ However, the decline in BMD in MM-induced osteoporosis has been less understood. In a recent study, Borggrefe et al^[17]^ found that BMD of fracture cases in MM patients were significantly reduced in men, but not in women. Hence, understanding the decline in BMD due to MM-induced osteoporosis may be unreliable. Second, BMD has been challenged as a limited tool for the diagnosis of osteoporosis itself as it only partially predicts fracture risk,^[18]^ which renders its use in the diagnostic evaluation of MM less valid. Also, routine assessment of BMD in MM patients has not been recommended due to methodological difficulties of this technique in these patients and the frequent use of bisphosphonates in all symptomatic MM patients.^[19]^ Third, there is no established clinical criterion in differentiating between osteoporotic VCFs and MM-induced osteoporotic VCFs.^[20]^

Finite element (FE) analysis, based on computed tomography (CT) imaging, is a noninvasive alternative to assess bone strength. FE analysis is a computational approach, where radiological scan data of patients are translated into three-dimensional (3D) models to predict structural behavior using numerical method.^[21]^ These patient-specific anatomical models are provided with appropriate material properties, boundary, and loading conditions that aims to mimic as closely the in vivo fracture conditions as possible, to obtain realistic predictions of the structural strength and other related properties, and to better understand the multifactorial etiology behind fractures for instance. The supremacy of CT-based FE analysis, for example, in predicting bone strength over the use of gold standard BMD, differentiating femoral strength due to various treatment options, and discerning patients with and without osteoporosis have been established in several studies.^[22–27]^

Therefore, this preliminary study aims to first validate FE analysis using experimentally determined failure load values obtained during an in vitro experiment performed on vertebra segments (T9–T12) from 3 fresh frozen human donors. This study also aims to apply the validated FE analysis to vertebra segments (T1–L5) modeled from in vivo multidetector computed tomography (MDCT) imaging scans from 4 MM patients with and without VCFs to assess individual fracture risk as well as correlate the fracture risk with MDCT-derived BMD measurements.

## 2 Materials and methods

### 2.1 Specimens and subjects

Fresh human vertebrae (n = 12; anatomical location: T9–T12) from 3 donors (one 74-year-old woman and two 46- and 62-year-old men, respectively) were obtained from the local Institute of pathology and anatomy. These donors were free of any skeletal diseases. The donors had dedicated their bodies for educational and research purposes to the local Institute prior to death, in compliance with local institutional and legislative requirements. Whole-body MDCT images of 4 subjects (one 60-year-old man and three 58-, 68-, and 71-year-old women, respectively) with new diagnosis of MM were retrospectively identified in our institution's digital image archive (PACS). The study was reviewed and approved by the local institutional review board (Ethikkommission der Fakultaet fuer Medizin der Technischen Universitaet Muenchen, Munich, Germany).

### 2.2 MDCT imaging

The in vitro vertebrae were scanned using a 256-row MDCT scanner (iCT, Philips, Netherlands.). Scan parameters were a tube voltage of 120 kVp, a tube load of 585 mAs, an image matrix of 1024 × 1024 pixels, and a field of view of 150 mm. Transverse sections were reconstructed with a high-resolution bone kernel (YE). The interpolated voxel size was of 146 × 146 × 300 μm^3^, while the real spatial resolution, as determined at q50 of the modulation-transfer-function, was 250 × 250 × 600 μm^3^. A dedicated calibration phantom (Mindways Osteoporosis Phantom, San Francisco, CA) was placed in the scanner mat beneath the vertebrae in all scans. In vivo MDCT imaging of patient with MM were performed with a 64-row MDCT scanner (Somatom Sensation 64, Siemens Healthcare, Erlangen, Germany). Scan parameters were a tube voltage of 120 kVp, an averaged tube current of 78 mA, an image matrix of 512 × 512 pixels, a pixel spacing of 977 × 977 μm^2^, a slice thickness of 670 μm, and a field of view of 150 mm. Transverse sections were reconstructed with a bone kernel (B70f). For calibration purposes, a reference phantom with a bone-like and a water-like phase (Osteo Phantom, Siemens Healthcare) was placed in the scanner mat beneath patients.

### 2.3 Biomechanical testing

Each vertebra was embedded in resin (Rencast Isocyanat and Polyol, Huntsman Group, Bad Säckingen, Germany) up to 2 mm above respectively below their vertebral endplates for the purpose of mechanical testing. The resin fixation was performed with parallel alignment of the upper and lower endplate of the vertebrae with the outer surface of the resin chock to guarantee strict axial loading conditions of the vertebrae during the uniaxial mechanical test. The resin-embedded vertebrae were fixed in a mechanical testing system (Wolpert Werkstoffprüfmaschinen AG, Schaffhausen, Switzerland). First, 10 preconditioning cycles with uniaxial tension-compression up to a load between 10 and 400 N with a rate of 5 mm/minute were applied. Then, a monotonic, uniaxial compression was performed at the same rate. The load–displacement curve was recorded and vertebral failure load was defined as the 1st peak of the load–displacement curve with a subsequent drop of >10%.

### 2.4 Finite element analysis of in vitro and in vivo models

**Figure 1:**
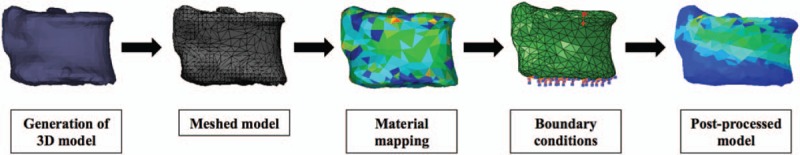
Flow diagram of finite element (FE) analysis of a typical vertebra segment.

FE analysis was applied for the 12 in vitro vertebrae samples as well as the in vivo MDCT whole-body scans of 4 MM patients obtained (n = 4). The DICOM images were imported into commercial software, Mimics (Materialise, Harislee, Belgium). 3D models of the in vitro vertebrae samples were immediately generated whereas, for each in vivo MDCT scan of MM patients, 3D models of the whole spine was 1st generated and then the thoracic and lumbar vertebrae (T1–L5) were segmented into individual segments (Fig. 1).

First, wrapping was applied to all vertebrae segments to reduce surface roughness that can result in meshing difficulties. This was followed by automatic meshing by 3-Matic (Materialise, Harislee, Belgium) using linear tetrahedral C3D4 elements with a constraint of a maximum aspect ratio of 25 (Fig. 1). Heterogeneous, nonlinear, and anisotropic material properties were assigned by discretizing the model into 10 different sets of materials.^[28]^ Cortical bone was simplified and assumed as denser trabecular bone;^[29]^ hence, only 1 set of relationships were used to calculate material properties as presented in Table 1. Negative modulus values resulting from low density regions were set to 0.0001 MPa.^[22]^ Material yield and ultimate failure were assumed to coincide, and a nonlinear postyield material behavior was adopted.^[30]^

**Table 1:**
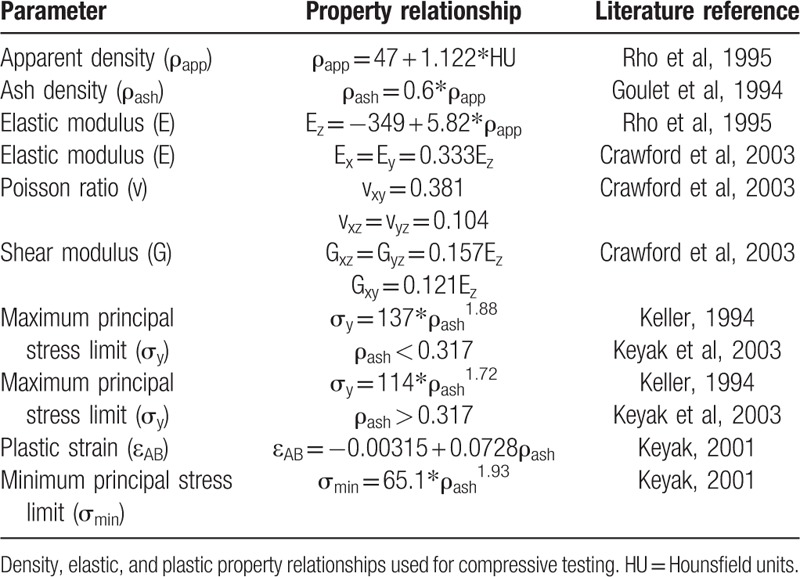
Material property relations adapted from literature.

FE analysis was performed using ABAQUS version 6.10 (Hibbitt, Karlsson, and Sorensen, Inc., Pawtucket, RI). Boundary conditions were applied to emulate axial compression in the vertical direction, as VCFs are the most common type of fractures resulting from compressive loadings. The displacement and rotation of the surface nodes on the inferior endplate was constrained in all directions, while a displacement load in the z-direction was applied incrementally on the surface nodes on the superior endplate (Fig. 1).^[31]^ The predicted fracture load was defined as the peak force of the force–displacement curve over the displacement increments. The predicted fracture load values were then compared with the experimentally measured values.

### 2.5 MDCT-derived BMD assessment

Elliptical regions of interest (ROIs) were placed on the each axial slice, with a slice thickness of 0.67 mm, for each vertebra (T1–L5) for each patient. The ROIs are placed such that they cover as big a diameter in the vertebra segment as possible, excluding any cortical bone.^[32]^ The mean and standard deviation of Hounsfield units (HUs) were calculated in each ROI. In addition, reference ROIs were also placed within 2 regions in the bone density calibration phantom composed of hydroxyapatite (HA), where the water-like part of phantom has HA density of 0 mg/cm^3^ (HA_w_), and the bone-like part of the phantom has HA density of 200 mg/cm^3^ (HA_b_). The HUs of these reference ROI regions were also measured on each slice; HU_w_ and HU_b_ representing the HUs for the water-like and bone-like regions, respectively. By assuming a linear relationship and by interpolating between the water-like and bone-like HUs, BMD was calculated by the following formula: BMD = [HA_b_/(HU_b_ − HU_w_)]∗(HU − HU_w_).^[33]^

### 2.6 Statistical analysis

Due to the small number of subjects analyzed in this study, pooled values of BMD, experimental, and FE-predicted vertebral strength values for all vertebra segments (n = 68) were compared and correlated. Unadjusted Spearman rank correlation coefficient values were calculated since it is resistant to outliers.^[34]^ The *P* values were considered significant if <0.05. All analyses were performed with a spreadsheet application (Microsoft Office Excel 2010, Redmond, WA).

## 3 Results

### 3.1 In vitro validation

**Figure 2:**
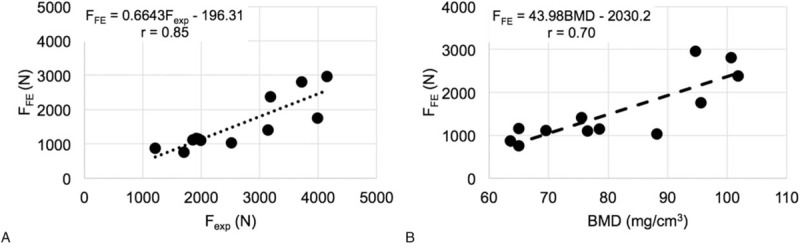
Plots of FE-predicted strength (F_FE_) as a function of experimentally determined strength (F_exp_) (A) and FE-predicted strength (F_FE_) as a function of BMD (B). BMD = bone mineral density, FE = finite element, F_FE_ = failure load values predicted from FE 55 models, F_exp_ = experimentally obtained failure load values.

Failure load values predicted from the FE models (F_FE_) of the in vitro vertebrae samples (n = 12) realistically matched experimentally obtained values (F_exp_), with a significant correlation of *r* = 0.85 (*P* < 0.001) (Fig. 2A). Also, Spearman rank correlation coefficient was also significant with *r* = 0.70 (*P* < 0.05) between F_FE_ and BMD (Fig. 2B), and *r* = 0.75 (*P* < 0.05) between F_exp_ and BMD.

### 3.2 In vivo finite-element analysis

**Figure 3:**
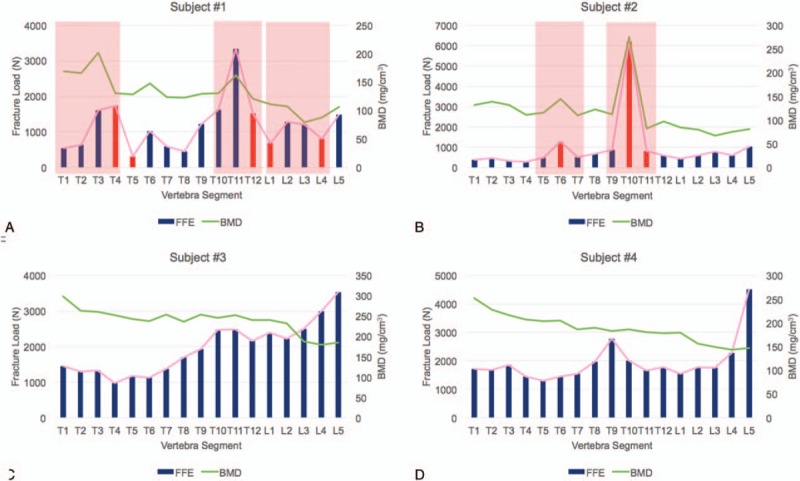
Patient-specific FE-predicted strength and BMD in each thoracic and lumbar vertebra segments (T1–L5) of subject #1 (A), subject #2 (B), subject #3 (C), and subject #4 (D). BMD = bone mineral density, FE = finite element.

**Figure 4:**
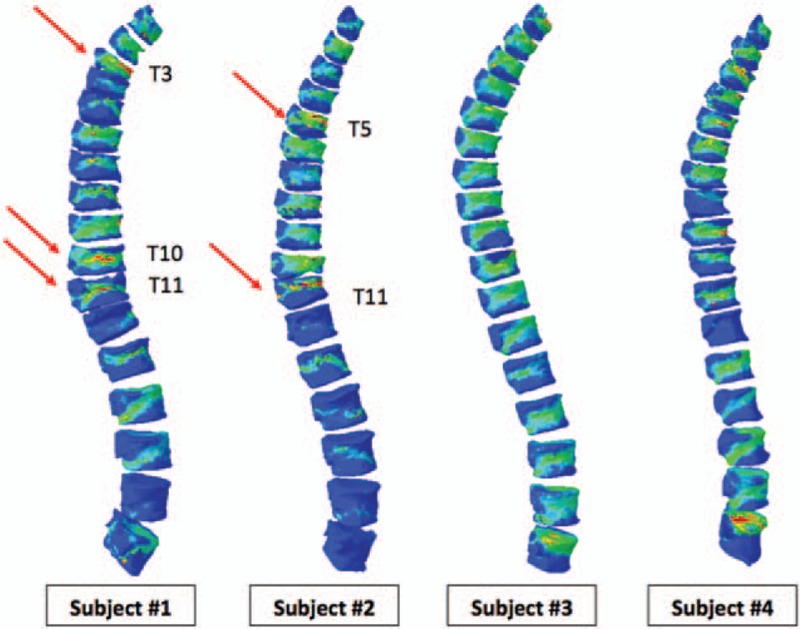
Maximum principal strain values from FE analysis of T1–L5 of each MM subject. Geometrically compromised segments exhibited higher maximum principal strain values, denoted by red regions. FE = finite element, MM = multiple myeloma.

FE-predicted strength values of each vertebra were studied in each patient (n = 4). There were several key findings obtained in this study. First, this study examined abrupt changes in fracture loads and discovered that subjects with fractures exhibited an erratic behavior in fracture loads between adjacent spinal segments. We characterized this instability by observing peaks in fracture load values highlighted in pink rectangular boxes while the fractured segments were denoted as red columns (Fig. 3). In subject #1, there were peaks associated with T3–T4 (peak 1), T11 (peak 2), and L2–L3 (peak 3) segments and in subject #2, there were peaks at T6 (peak 1) and T10 (peak 2). Subject #1 had originally attained fractures at the T4, T5, T12, L1, and L4 segments. Consequently, it was indicative that segments adjacent to these peaks seemed to also experience regions of instability. Hence, the 2nd finding was that peaks in fracture load seem to place the peak-associated segments as well as the adjacent segments at risk of fracture. Similarly, for subject #2, adjacent segments at risk were T5, T7, T9, and T11. This corresponded to fractured segments attained by subject #2, at T6, T10, and T11. Third, subjects without fractures exhibit gradual changes in FE-predicted fracture load values. In subject #3 and subject #4, no peaks were observed, indicating a low risk of fracture. Third, the existence of peaks were also further quantified by calculating the relative changes of fracture loads of each segment, with respect to its following adjacent segment, for example, T1 with respect to T2, and T2 with respect to T3 (Table 2). The higher the relative change, the greater the instability locally and for this preliminary study, the relative change was considered to be unstable when it exceeds a value of 1.00. The vertebrae segments highlighted were T4, T11, and T12 in subject #1 and T6 and T10 in subject #2 (Table 2). To place this finding into perspective, Table 3 shows the peak-associated segments at risk, adjacent segments at risk, and fractures attained by subject #1 and subject #2. All fractures attained by subject #1 and subject #2 were identified as either a peak-associated segment or adjacent segment at risk. Last, it was also observed that geometrically compromised segments exhibited higher maximum principal strain values (denoted by red regions) (Fig. 4). In subject #1 and subject #2, T3, T10 and T11, and T5 and T11 showed critical plastic strain regions, respectively, whereas in subject #3 and subject #4, the segments showed geometric stability and insignificant critical strain regions.

**Table 2:**
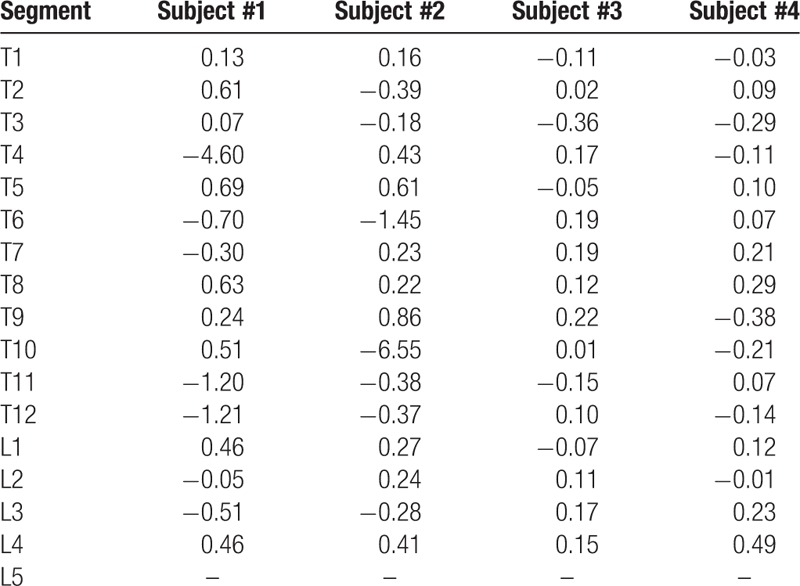
Relative changes of fracture loads of each segment with respects to following adjacent segment (values greater than 1.00 denoted in red).

**Table 3:**
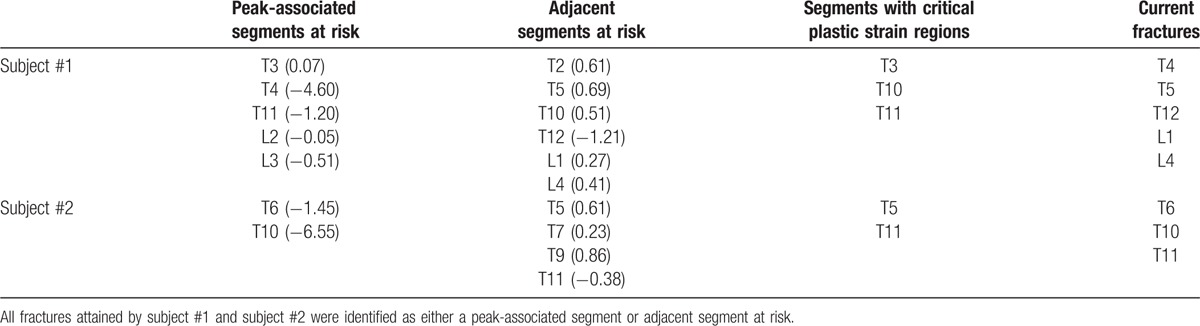
Peak-associated segments at risk, adjacent segments at risk, segments with critical plastic strain regions, and current fractures attained by subject #1 and subject #2.

### 3.3 MDCT-derived BMD assessment

The Spearman rank correlation coefficient was *r* = 0.79 (*P* < 0.001) for the correlation between F_FE_ and BMD for lumbar segments (L1–L5) and *r* = 0.58 (*P* < 0.001) for thoracic segments. The pooled coefficient for all the vertebrae segments was *r* = 0.57 (*P* < 0.001).

## 4 Discussion

MM is still not a well-understood skeletal disease, although it poses significant burden to the society, especially being a prevalent condition among the elderly. This study showed that by applying the same universal loading condition to the vertebra segments from T1 to L5, the differences in structural strength could be compared within each patient and between patients. Thus, the assessment of the biomechanical properties of vertebrae segments can be performed with noninvasive CT-based FE modeling. Applying FE methodology in the clinical scenario will enable clinicians to look at all the spine segments concurrently to analyze the structural strength differences and in this case better predict vertebral fracture risk in MM. The accuracy of the FE modeling was validated by performing experimental testing with human cadaveric specimens. Vertebral bone strength was accurately estimated by applying the FE modeling protocol.

**Figure 5:**
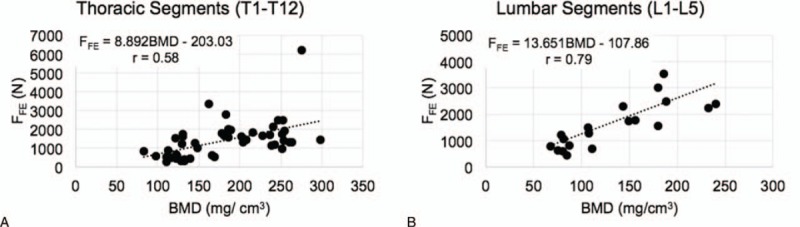
Plots of FE-predicted strength (F_FE_) as a function of BMD in thoracic vertebrae (A) and lumbar vertebrae (B) of MM subjects. BMD = bone mineral density, FE = finite element, F_FE_ = failure load values predicted from FE 55 models, MM = multiple myeloma.

A novel aspect of this study is the use of MDCT and FE-based structural strength assessment to assess relative vertebral fracture risk within vertebra segments in each subject. Although the correlation between FE-predicted strength and MDCT-derived BMD was moderate (Fig. 5), the interesting finding was on the erratic nature of fracture load values found in patients who have already attained fractures (ie, subject #1 and subject #2), placing the peak-associated segments and adjacent segments at high risk to future fractures (Fig. 3). This could be attributed to the fact that fractured segments have smaller than original geometries, resulting in extremely high bone density within a smaller volume per se, resulting in over-prediction of their fracture loads. Also, they may have altered biomechanical properties that evades away from material relations found in current literature for osteoporosis. This is further exacerbated by influences of MM itself on the bone characteristics, which could also explain the moderate correlation obtained between FE-predicted strength and BMD.

**Figure 6:**
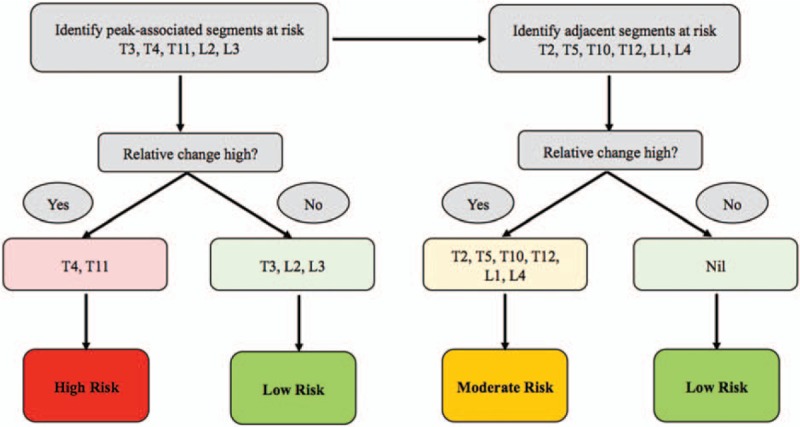
Schematic illustration of patient-specific risk assessment strategy (subject #1 applied as an example).

Nevertheless, with information on fracture load values, relative changes in fracture loads as well as geometric, and critical plastic strain region observations, clinicians could utilize a guiding tool to evaluate patient-specific fracture risk of the spine in MM patients (Fig. 6). A schematic on how patient-specific risk assessment could be evaluated is illustrated. By first identifying the peak-associated segments at risk, the high relative change segments can be categorized under high risk, while for the segments with moderate/low relative change under low risk. Following that, adjacent segments at risk need to be identified, categorizing them similarly as aforementioned into moderate and low risk categories (Fig. 6). In the case of subject #1, it is evident that regions of severe structural instability were exhibited throughout the individual segments of the spine. This phenomenon suggests that the whole spine of subject #1 was at risk of fracture. Evaluation of more MM subjects would enable us to have a more accurate patient-specific risk assessment and treatment strategy.

This study also suggests that absolute value of fracture loads may have little value and it is the relative fracture loads that will provide valuable information on the relative stability between segments. Most studies have presented absolute values in one or more vertebral osteoporotic segments, but this is the first study of its kind to report the fracture load values that can be simultaneously observed across the vertebrae segments in each patient. In early diagnosis, the presence of peaks could indicate regions of low structural stability, providing the clinicians with preemptive treatment options to prevent the occurrence of fractures. This is valuable information to the clinicians as they will be able to prescribe more targeted drug therapy^[35]^ as well as exercise regimes that improve the structural stability of the spine at the affected areas.^[36]^ Furthermore, fracture load values provide amplified differences between segments, compared to BMD, making it easier to identify segments at high fracture risk.

Since MM is a plasma disease, it may result in severe bone loss due to crosstalk between cells responsible for bone cell activity, which explains the significantly low range of fracture loads obtained. A study showed that in osteoporotic patients over the age of 65, the threshold strength in the spine for fragile bone that is highly susceptible to risk of fracture is 4500 N for women and 6500 N for men.^[37]^ In this preliminary study of n = 4 subjects, we found that the range of fracture load for subject #1 (male) was 310 to 3340 N, whereas for subject #2, it was 284 to 1274 N. Studies have shown that women aged over 50 years exhibited high correlation between vertebral strength and load-to-strength ratio to prevalent vertebral fractures.^[38–40]^ These collective literature findings also support the findings in this study that vertebral strength can be used as a diagnostic tool to access the risk of vertebral fractures.^[37]^

Although the degeneration of the spine due to the onset of MM has not been well understood, osteoporosis of the spine has been sufficiently studied by researchers. Fragility fractures have been understood to be loss in structural strength due to the weakening of the trabecular structure and an under compensation of bone resorption in the bone remodeling process, that leads to lower bone turnover.^[41]^ However in MM, the mechanisms are incompletely understood. Due to increased receptor activator of nuclear factor kappa-B ligand production by the bone marrow stromal cells, and an increased degradation of osteoprotegerin, there is increased osteoclast precursor differentiation and consequently, enhanced bone resorption.^[42]^ MM-derived mesenchymal stem cells produce osteoblasts that are different from healthy osteoblasts produced by normal mesenchymal stem cells. This may indicate that the progression of MM is independent of osteoporosis, which may be a by-product of the bone carcinoma. It is not clear how osteoporosis influences this bone cancer and vice versa. Also, it is unclear how to explain the nonspecific changes that occur due to underlying biological mechanisms in MM patients, but it seems apparent that MM can result in significant reduction in structural strength, causing certain adjacent segments to have fluctuating strengths.

This study has several limitations. First, this preliminary study only examined 4 MM subjects, 2 with and 2 without fractures. Not only will a bigger sample enable statistical emphasis on the findings, a threshold value for the relative change between vertebra segments could be evaluated too. This threshold value will serve as a better clinical indication on the risk of fracture and consequently aid in predicting the occurrence of fractures. Also, the resolution of scans used for in vitro validation was higher than that of the scans used for in vivo scans of MM subjects, due to the restrictions imposed on radiation exposure. Thirdly, we analyzed one simple compressive loading condition, but there is a combination of loadings occurring in vivo, for example flexion, rotation, and bending. However, axial compressive loading accounts for 90% of fracture cases experienced by clinicians. Nevertheless, future work will entail using combination loading conditions to more accurately predict fractures in MM patients. Only fracture load values were used as an assessment parameter. Future study will also look into using geometric parameters such as buckling ratio, which has been proven to indicate structural stability.

In conclusion, this study found that structural instability between vertebra segments could be the first tell-tale sign of impending fractures. The erratic nature of fracture load values between the segments in patients who have attained fractures is evidence that examining the stability of the vertebrae segments in a patient specific manner could enable early prediction of fractures in MM patients. Further study on establishing a threshold value for the relative change in fracture load values between adjacent segments could be useful for clinicians to identify segments at risk and suggest targeted treatment to the affected segments before the occurrence of fractures. This noninvasive patient-specific examination will serve to provide key information on the relative stability of the vertebra segments in MM patients, reducing the morbidity and mortality of elderly diagnosed with MM in the long run.
