# Risk of vertebral compression fractures in multiple myeloma patients: A finite-element study: Erratum

**DOI:** 10.1097/MD.0000000000007017

**Published:** 2017-05-19

In the article, “Risk of vertebral compression fractures in multiple myeloma patients: A finite-element study”,^[1]^ which appeared in Volume 96, Issue 2 of *Medicine*, two authors’ names, degrees and affiliations appeared incorrectly. Baum Thomas, PhD should appear as Thomas Baum, MD. This author's affiliation should be “Department of Neuroradiology, Klinikum rechts der Isar, Technische Universitaet Muenchen, Muenchen, Germany.” Kirschke S. Jan, PhD should appear as Jan S Kirschke, MD. This author's affiliation should be “Department of Neuroradiology, Klinikum rechts der Isar, Technische Universitaet Muenchen, Muenchen, Germany.”
